# A Novel Technique for Simultaneous Double Annular Enlargement Using a Single Dacron Graft

**DOI:** 10.1016/j.atssr.2025.12.001

**Published:** 2025-12-20

**Authors:** Majed Tolah, Dragan Opacic, Mohammad Sharaf, Joanna Gilis-Januszewski, André Renner, Jan Gummert, Tomasz Gilis-Januszewski

**Affiliations:** 1 Department of Cardiothoracic Surgery, Heart and Diabetic Center of North Rhine-Westphalia, Bad Oyenhausen, Germany; 2 Department of Cardiac Surgery, Madinah Cardiac Center, Madinah, Saudi Arabia

## Abstract

**Purpose:**

We describe a novel surgical technique for simultaneous enlargement of both the aortic and mitral annuli using a single Dacron (DuPont) graft.

**Description:**

The mitral prosthesis is first sutured within the graft, followed by trimming and fixation of the aortic prosthesis at a predetermined offset. The completed conduit is then implanted en bloc, allowing controlled, symmetric annular enlargement with precise geometric alignment.

**Evaluation:**

The technique was applied in a redo case after prior aortic valve replacement with a Freestyle (Medtronic) prosthesis in a patient presenting with prosthetic stenosis and mitral valve endocarditis. The procedure simplified complex reconstruction, reduced operative steps, avoided multiple patches, and preserved the geometry of the annuli and left ventricular outflow tract. The patient recovered uneventfully with excellent postoperative hemodynamics.

**Conclusions:**

This streamlined approach offers a practical and reproducible alternative for complex double-valve replacement with concomitant aortic root replacement involving the fibrous skeleton, particularly in patients with small annuli, heavy calcification, porcelain aorta, or prior surgery.

## Visual Abstract

**Figure:**
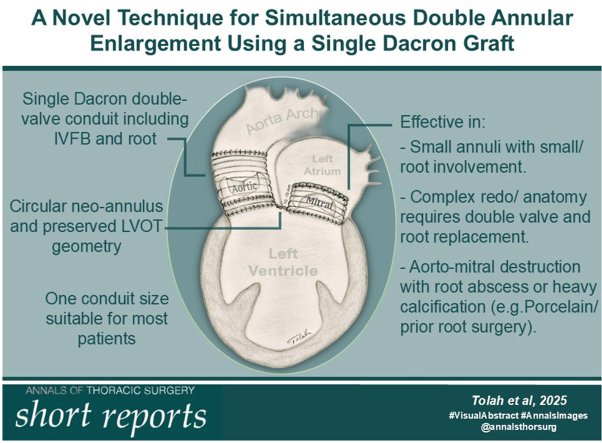

## Technology

The “Commando” procedure, first described by David et al^1^ in 1997, addresses complex pathologies of the left fibrous skeleton by reconstructing the intervalvular fibrous body. Although effective, it is technically demanding and poorly suited for patients with small aortic and mitral annuli. Over time, several modifications have emerged.^2^^,^^3^ Here, we introduce a new simplified technique using a single Dacron (DuPont) graft to simultaneously enlarge both annuli and provide a safe, reproducible, and efficient alternative in which one conduit size fits all patients.

## Technique

**Figure 1:**
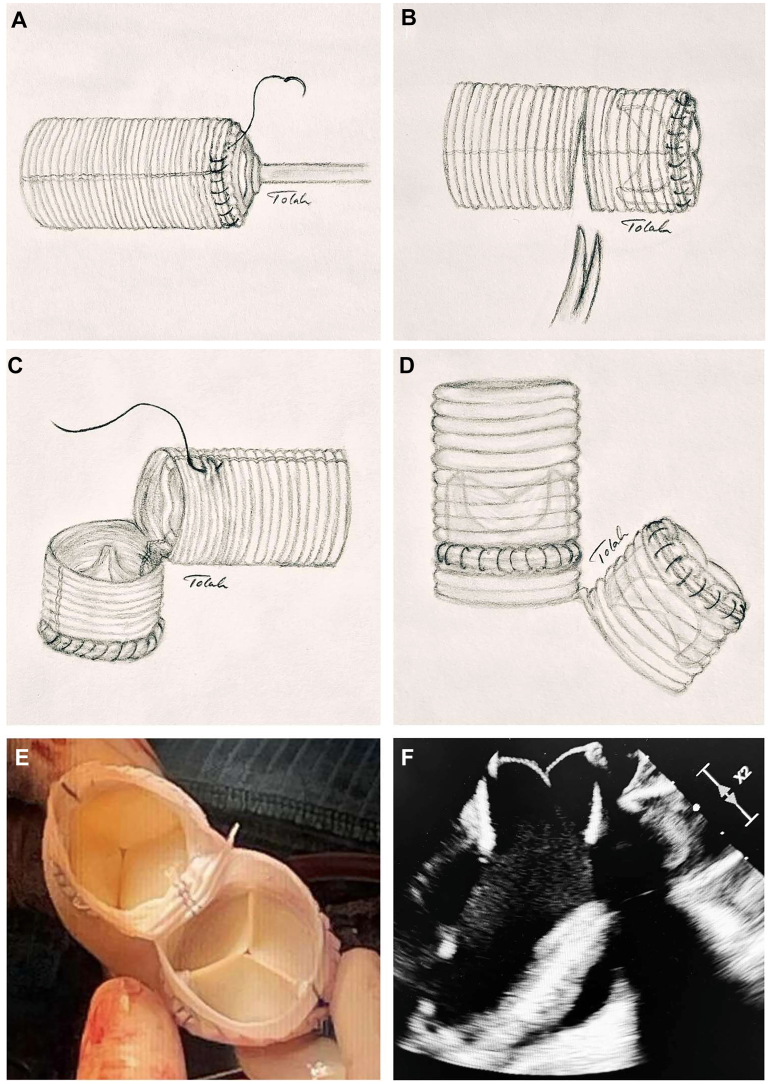
Conduit preparation: (A) Mitral prosthesis is sewn into the Dacron graft. (B) Two-thirds of the graft us trimmed above the struts. (C) The aortic prosthesis inserted and sutured 5 mm from the edge. (D) Final conduit with both prostheses. (E) Surgical and (F) echocardiographic views.

After resternotomy and careful adhesiolysis, aortic and bicaval cannulation was performed. Before cardiopulmonary bypass was initiated, the conduit was prepared (Figure 1). A 29-mm mitral prosthesis (Perimount Magna Ease, Edwards Lifesciences) was sewn to the end of a 32-mm Dacron graft (Hemashield, Getinge) using a 4-0 Prolene (Ethicon) running suture. Two-thirds of the graft circumference was trimmed just above the valve struts, and the graft was bent. A 27-mm aortic prosthesis (Perimount Magna Ease) was then inserted 5 mm above the trimmed edge and secured with a 4-0 Prolene suture. Pledgeted 4-0 Prolene sutures were inserted along the folding line of the conduit. These sutures were later used to close the left atrial roof. Valve and conduit sizes were selected for broad anatomical compatibility and depending on the opening orifice and the patient’s body mass index and body surface area.

After the conduit was prepared, cardiopulmonary bypass was initiated and the aorta was cross-clamped. The aortotomy was extended toward the aortomitral curtain, reaching the anterior mitral leaflet, followed by left atrial dome opening. Intraoperative findings revealed extremely small aortic annuli and subannular diameters, along with small infected mitral valve. The Freestyle (Medtronic) cusps and anterior mitral leaflet were excised. For better surgical exposition, the superior vena cava was mobilized.

**Figure 2:**
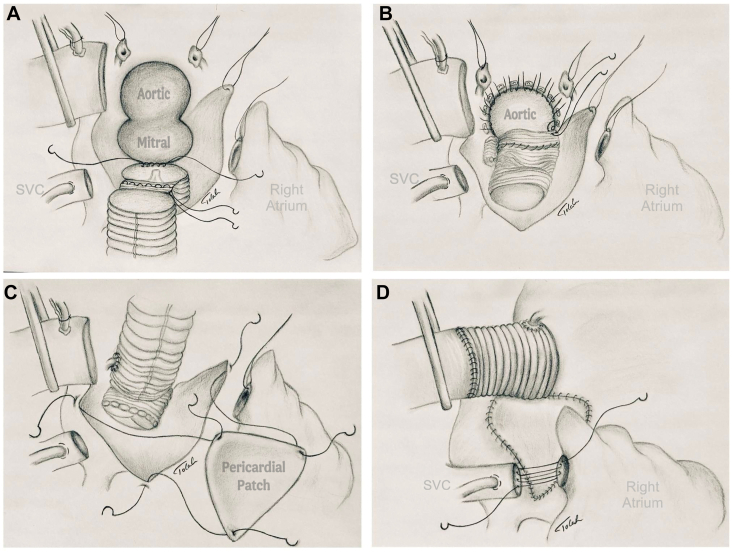
Conduit implantation: (A) Mitral valve is sutured; atrial closure sutures are placed. (B) Aortic valve is implanted. (C) Left atrium is closed with a pericardial patch. (D) Conduit is connected to the aorta, and superior vena cava (SVC) is reattached.

**Figure 3:**
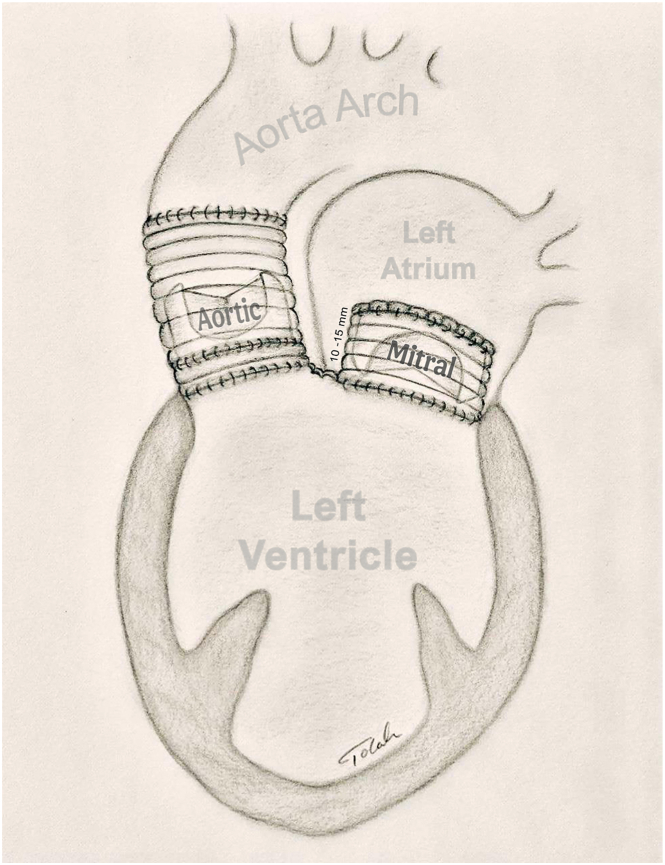
Anatomical cutdown view of the conduit containing both aortic and mitral valves.

The conduit (Figure 2) was implanted starting with the mitral valve using a 2-0 Prolene running suture from P2 to A1, and then the other side was sewn in the other direction toward A3. The aortic portion was anchored into the left and right coronary sinuses using 2-0 Ethibond (Ethicon) pledgeted interrupted sutures. The severely altered coronary buttons prevented a typical attachment to the aortic root; therefore, the Cabrol technique was used for coronary reimplantation. The left atrium was closed with a pericardial patch, and the prepared interrupted sutures were used to close the roof adjacent to the graft. Finally, the conduit was distally connected to the aorta, and the superior vena cava was reattached (Figure 3). Surgical details are further illustrated in the Video

This technique was applied in a 61-year-old woman with severe aortic prosthesis stenosis and mitral regurgitation. She had undergone AVR with a 23-mm Freestyle valve in 2011 and had recently been treated for mitral endocarditis. The Herz und Diabeteszentrum–North Rhine-Westphalia Institutional Review Board approved the study (No. 2025-1401, 02.07.2025), and written informed consent was secured from the patient.

The patient’s postoperative course was uneventful. Echocardiography showed well-functioning prostheses, with a mean gradient of 6 mm Hg aortic and 1.5 mm Hg mitral. The patient was discharged in stable condition and followed up at 6 months.

## Clinical Experience

Various surgical techniques have been developed to reconstruct the fibrous skeleton, yet each carries limitation. The Commando procedure is technically demanding and poorly suited for patients with small aortic or mitral annuli. A key drawback is distortion of aortic annular geometry after mitral annular enlargement, which can restrict mitral prosthesis sizing.

Incision of the aortomitral curtain and anterior mitral extension disrupts semilunar commissure balance, transforming the circular aortic root and left ventricular outflow tract (LVOT) into an oval shape. This distortion increases the risk of LVOT obstruction, especially near the left/noncoronary cusp, and complicates secure implantation of a round prosthesis in the aortic position, particularly in small anatomies. Dual pericardial patches and precise positioning of 2 prostheses are also required, raising the risk of paravalvular leaks and other complications.

Modifications such as the chimney technique (Yang and colleagues^3^) mitigate LVOT obstruction by anchoring only the mitral prosthesis to the conduit. This reduces valve interference, although the aortic prosthesis remains within a distorted, possibly small annulus, causing prosthesis-patient mismatch. Yang and colleagues^4^ later refined this approach with a Y-incision annular enlargement. David and colleagues^2^ introduced a dual-graft technique, connecting each prosthesis to separate grafts; although solving the issue of valve interference, it further increased surgical complexity.

Our technique, using a single Dacron graft for simultaneous enlargement of both annuli with concomitant aortic root replacement, addresses these limitations. It creates a standardized, circular neoannulus that reduces geometric distortion, mechanical tension, and the need for dual patches. Prostheses are seated within a defined geometry, improving alignment and simplifying implantation.

Concerns addressing the risk of blood stagnation and thrombus formation rise after such surgical intervention. Although our postoperative echocardiographic images did not show any signs of blood stagnation, we still recommend the anticoagulation with warfarin for at least 6 months, with a goal international normalized ratio of 2.5 to 3.5, and then non–vitamin K oral anticoagulants for patients with biological valves and a lifelong warfarin therapy with the same international normalized ratio goal for patients with mechanical valves.

This method is applicable in complex anatomies requiring double-valve replacement with concomitant aortic root replacement, including small annuli with small root, endocarditis with aortomitral destruction and root abscesses, extensive calcification, such as porcelain aorta and prior root surgery (eg, Freestyle or root enlargement). The valve sizes used also facilitate future valve-in-valve transcatheter aortic valve implantation or transcatheter aortic valve replacement.

## Comment

Prebypass conduit preparation enhances surgical efficiency. Compared with existing methods, this technique uses a single conduit to anchor both prostheses and graft, streamlining the procedure and minimizing valve interference compared with chimney.

## Disclosures and Freedom Of Investigation

The tested surgical technique was developed and performed independently by the authors. The authors had full control of the study design, methods used, data analysis, outcome variables and results, analysis of data, and production of the written report.

## Disclaimer

The Society of Thoracic Surgeons, the Southern Thoracic Surgical Association, and *The Annals of Thoracic Surgery Short Reports* neither endorse nor discourage use of the new technology described in this article.
